# Left Ventricular Deformation in Patients with Connective Tissue Disease: Evaluated by 3.0T Cardiac Magnetic Resonance Tissue Tracking

**DOI:** 10.1038/s41598-019-54094-1

**Published:** 2019-11-29

**Authors:** Jin Wang, Ke Shi, Hua-yan Xu, Qin Zhao, Xi Liu, Yue Gao, Hong Yu, Ying-kun Guo, Zhi-gang Yang

**Affiliations:** 1 Department of Radiology, West China Hospital, Sichuan University, 37# Guo Xue Xiang, Chengdu, Sichuan 610041 China; 2 Department of Radiology, Key Laboratory of Birth Defects and Related Disease of Woman and Children of Ministry of Education, West China Second University Hospital, Sichuan University, Chengdu, China

**Keywords:** Connective tissue diseases, Cardiovascular diseases, Cardiovascular diseases

## Abstract

The aim of this study was to assess left ventricular (LV) myocardial strain in patients with connective tissue disease (CTD) and compare LV deformation between subgroups of idiopathic inflammatory myopathy (IIM) and non-IIM. Ninety-eight patients with CTD, comprising 56 with IIM and 42 with non-IIM, and 30 healthy subjects were enrolled and underwent 3.0T cardiac magnetic resonance imaging (MRI) scanning. The LV function and strain parameters were measured and assessed. Our result revealed that CTD patients had preserved LV ejection fraction (60.85%) and had significantly decreased global and regional peak strain (PS) in radial, circumferential, and longitudinal directions (all *p* < 0.05). IIM patients showed significantly reduced global longitudinal PS (GLPS) and longitudinal PS at apical slice, whereas all strain parameters decreased in non-IIM patients. Except GLPS and longitudinal PS at apical slice, all strain parameters in non-IIM patients were lower than those in IIM patients. By Pearson’s correlation analysis, the LV global radial and circumferential PS were correlated to N-terminal pro-brain natriuretic peptide level and LV ejection fraction in both IIM and non-IIM patients. This study indicated that CTD patients showed abnormal LV deformation despite with preserved LVEF. The impairment of LV deformation differed between IIM and non-IIM patients.

## Introduction

Connective tissue disease (CTD), including systemic lupus erythematosus (SLE), rheumatoid arthritis (RA), idiopathic inflammatory myopathy (IIM), and systemic sclerosis (SSc), represent a heterogeneous group of inflammatory diseases derived from an auto-immunological deregulation and characterized by multiorgan involvement^1,2^

Cardiovascular complications in patients with CTD are among the leading causes of death^3^. Given that the cardiovascular manifestations in CTD can be clinically non-specific and insidious^2^, it is imperative for physicians to detect cardiac involvement early, before subclinical left ventricular (LV) dysfunction progresses to irreversible heart failure.

IIM is characterized by muscle weakness and inflammatory cell infiltration in skeletal muscle^4^. As the myocardium is a modified skeletal muscle, possibilities are that the immune-mediated inflammation also acts in myocardial disease in patients with IIM^5^. In other CTD, the underlying mechanisms of myocardial involvement are not fully understood; while, consequent autoantibody-related injury in the myocardium has been reported^6^.

Cardiac magnetic resonance imaging (MRI) tissue tracking has recently been developed for the assessment of myocardial deformation and subclinical cardiac dysfunction prior to a reduction in the LV ejection fraction (LVEF)^7,8^. However, to the best of our knowledge, few studies have evaluated myocardial dysfunction in patients with CTD using cardiac MRI tissue tracking^9,10^. Furthermore, there exists limited information regarding the differences in LV deformation between IIM and other CTD. Therefore, the present study aimed to quantitate the V myocardial systolic strain in patients with CTD and investigate whether the LV myocardial deformation was different between patients with IIM and non-IIM.

## Results

### Patient characteristics

All basic characteristics are summarized in Table 1.Patients with non-IIM were mainly females. Among the three groups, normal controls, IIM, and non-IIM, N-terminal pro-brain natriuretic peptide (NT-proBNP) was gradually increased [69 (31, 90) vs. 119 (57, 306) vs. 1272 (266, 5047), *p* < 0.05]. In patients with CTD, LVEF was preserved (LVEF > 50%) in both the IIM [63.9 (56.9, 67.9)] and non-IIM [56 (40.9, 62.5)] group. The LV function parameters, including LV end-diastolic volume (LVEDV), LV end-systolic volume (LVESV), LV stroke volume (LVSV), showed no differences between the normal controls and patients with CTD (all *p* > 0.05).

**Table 1:** Baseline characteristics. Notes: Values are presented as the mean ± SD, n (%), or median (quartile).

	Normal controls	CTD	CTD	
	(n = 30)	(n = 98)	IIM (n = 56)	non-IIM (n = 42)
Age(years)	45.5 ± 12.3	45.2 ± 13.2	47.9 ± 13.2	43.8 ± 13.0
Male(n,%)	11 (36.7%)	24 (24.5%)	20 (35.7%)	4 (9.5%)^*§^
BMI (Kg/m^2^)	20.8 ± 1.3	21.5 ± 2.8	21.8 ± 2.8	21.2 ± 2.7
BSA (m^2^)	1.6 ± 0.1	1.6 ± 0.2	1.6 ± 0.1	1.5 ± 0.1
Disease duration (years)	—	1.39 (1.0, 5.9)	1.2 (1.1, 1.8)	3.5 (0.5, 11.0)
Systolic blood pressure (mmHg) (mmHg)	116.7 ± 5.2	120.4 ± 16.9	122.0 ± 15.0	118.2 ± 19.0
Diastolic blood pressure (mmHg)	76.1 ± 5.3	79.2 ± 13.7	79.2 ± 13.3	79.2 ± 14.3
Heart rate (bpm)	74.9 ± 4.9	73.1 ± 9.6	72.5 ± 9.5	74.0 ± 9.8
NT-proBNP (pg/ml)	69 (31, 90)	214 (63, 1154)	119 (57, 306)^*^	1272 (266,5047)^*§^
LVEDV (ml)	122.1 (104.0, 137.7)	118.3 (96.2, 141.3)	117.1 (99.4, 138.7)	121.9 (91.7, 158.5)
LVESV (ml)	44.2 (38.9, 53.4)	45.8 (36.7, 63.4)	44.7 (36.5, 52.2)	52 (37.4, 88.4)
LVSV (ml)	77.4 (65.7, 82.8)	67.4 (53.9, 81.1)	69.8 (61.2, 86.1)	59.4 (50.6, 76.9)^*§^
LVEF (%)	61.5 (58.3, 64.5)	60.9 (52.2, 65.4)	63.9 (56.9, 67.9)	56 (40.9, 62.5)^*§^

^*^P < 0.05 versus normal controls; ^§^*P* < 0.05 versus patients with IIM. CTD, connective tissue disease; IIM, idiopathic inflammatory myopathy; BSA, body surface area; BMI, body mass index; NT-proBNP, N-terminal pro-brain natriuretic peptide; LV, left ventricular; LVEDV, LV end-diastolic volume; LVESV, LV end-systolic volume; LVSV, LV stroke volume; LVEF, LV ejection fraction.

### Comparison of the LV strain parameters between patients with CTD and normal controls

The global and regional strain parameters of all subjects are shown in Table 2. Compared with normal controls, the magnitude of global radial, circumferential, and longitudinal peak strain (PS) was decreased in patients with CTD [GRPS, 30.9 ± 7.6 vs. 23.8 ± 9.5%, *p* < 0.001; GCPS, −20.9 ± 1.9 vs. −18.5 ± 5.1%, *p* = 0.019; GLPS, −10.4 ± 3.2 vs. −8.3 ± 2.8%, *p* = 0.001].The magnitude of regional PS in all three directions was also significantly decreased (all *p* < 0.05).

**Table 2:** Comparison of LV strain parameters between normal controls and patients with CTD.

	Normal controls	CTD	CTD	
	(n = 30)	(n = 98)	IIM(n = 56)	non-IIM (n = 42)
**Global**				
Radial PS (%)	30.9 ± 7.6	23.8 ± 9.5^#^	26.8 ± 8.1	19.7 ± 9.7^*§^
Circumferential PS (%)	−20.9 ± 1.9	−18.5 ± 5.1^#^	−20.5 ± 3.7	−15.8 ± 5.4 ^*§^
Longitudinal PS (%)	−10.4 ± 3.2	−8.3 ± 2.8^#^	−8.6 ± 2.7^*^	−8.0 ± 2.9^*^
**Basal**				
Radial PS (%)	46.8 ± 9.7	37.9 ± 14.6^#^	41.8 ± 12.6	32.6 ± 15.5 ^*§^
Circumferential PS (%)	−16.8 ± 2.4	−15.3 ± 4.6^#^	−17.1 ± 3.7	−12.9 ± 4.7 ^*§^
Longitudinal PS (%)	−10.0 ± 3.5	−8.0 ± 3.6^#^	−8.9 ± 3.3	−6.8 ± 3.5^*§^
**Mid**				
Radial PS (%)	32.1 ± 8.8	24.6 ± 11.0^#^	27.9 ± 9.6	20.2 ± 11.2^*§^
Circumferential PS (%)	−21.3 ± 2.2	−18.3 ± 5.2^#^	−20.3 ± 3.7	−15.6 ± 5.7 ^*§^
Longitudinal PS (%)	−12.2 ± 3.2	−9.9 ± 3.8^#^	−10.8 ± 3.6	−8.7 ± 3.8^*§^
**Apical**				
Radial PS (%)	19.1 ± 11.0	14.6 ± 8.1^#^	17.0 ± 8.0	11.3 ± 7.0 ^*§^
Circumferential PS (%)	−24.9 ± 2.7	−22.2 ± 6.2^#^	−24.5 ± 4.8	−19.2 ± 6.7 ^*§^
Longitudinal PS (%)	−10.9 ± 4.9	−8.2 ± 3.6^#^	−8.1 ± 3.0^*^	−8.4 ± 4.4 ^*^

Notes: Values are presented as the mean ± SD . ^*^P < 0.017 versus normal group; ^§^*P* < 0.017 versus patients with IIM; ^#^*P* < 0.05 patients with CTD versus normal group. CTD, connective tissue disease; IIM, idiopathic inflammatory myopathy; PS, peak strain.

### Comparison of the LV strain parameters among IIM and non-IIM patients and normal controls

In contrast to the healthy normal subjects, the magnitude of GLPS (−10.4 ± 3.2% vs.−8.6 ± 2.7%, *p* = 0.006) and longitudinal PS at the apical slice [−10.9 ± 4.9% vs.−8.1 ± 3.0%, *p* = 0.012] were significantly reduced in the IIM group, whereas the magnitude of all global and regional strain parameters in the three directions were decreased in the non-IIM group (all *p* < 0.05). In the non-IIM group, the magnitude of all strain parameters in the radial, circumferential, and longitudinal directions were lower than those in the IIM group, with the exception of GLPS and longitudinal PS at the apical slice (all *p* < 0.05) (see Table 2 and Supplementary Fig. S1).

Receiver operating characteristic (ROC) analysis identified GRPS [cutoff value: 20%, area under the curve (AUC): 0.71, sensitivity: 59.52%, specificity: 82.14%], GCPS (cutoff value: −19.87%, AUC: 0.759, sensitivity: 76.19%, specificity: 64.29%) and GLPS (cutoff value: −9.85%, AUC: 0.55, sensitivity: 83.33%, specificity: 33.93%) to differentiate IIM from non-IIM.

### Association between LV global strain parameters and NT-proBNP level

**Figure 1:**
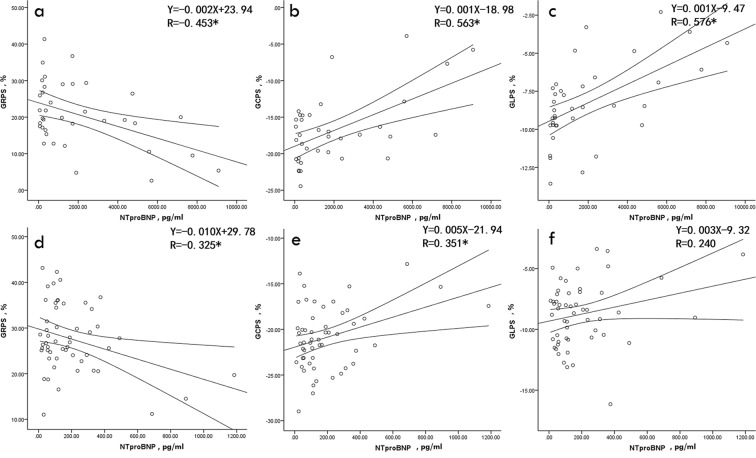
Pearson’s correlation analysis of Nt-proBNP with GRPS, GCPS, and GLPS in the non-IIM group (**a–c**) and IIM group (**d**–**f**). Notes: *P < 0.05. Actual *p* value and 95% CI are provided from a–f as follows: a (95% CI: −0.677, −0.151, *p* = 0.005), b (95% CI: 0.292, 0.750, *p* < 0.001), c (95% CI: 0.310, 0.758, *p* < 0.001), (**d**) (95% CI: *−*0.548, −0.060, *p* = 0.017), (**e)** (95% CI: 0.090, 0.568, *p* = 0.01), and (**f**) (95% CI: −0.033, 0.479, *p* = 0.084). CI: confidence interval; NT-proBNP, N-terminal pro-brain natriuretic peptide; GRPS, global radial peak strain; GCPS, global circumferential peak strain; GLPS, global longitudinal peak strain; IIM, idiopathic inflammatory myopathy.

In the non-IIM group, GRPS was negatively associated with NT-proBNP level (r = −0.453, *p* = 0.005) and GCPS and GLPS were positively correlated with NT-proBNP level (r = 0.563 and 0.576, respectively; both *p* < 0.001). In the IIM group, NT-proBNP was correlated with GRPS (r = −0.325, *p* = 0.017), as well as GCPS (r = 0.351, *p* = 0.01) and there was no correlation between NT-proBNP and GLPS (r = 0.240, *p* = 0.084) (see Fig. 1). There were no significant correlations between global strain parameters in the three directions and NT-proBNP level in the healthy volunteers (all *p* > 0.05) (see Supplementary Fig. S2a−c).

### Association between LV global strain parameters and LVEF

**Figure 2:**
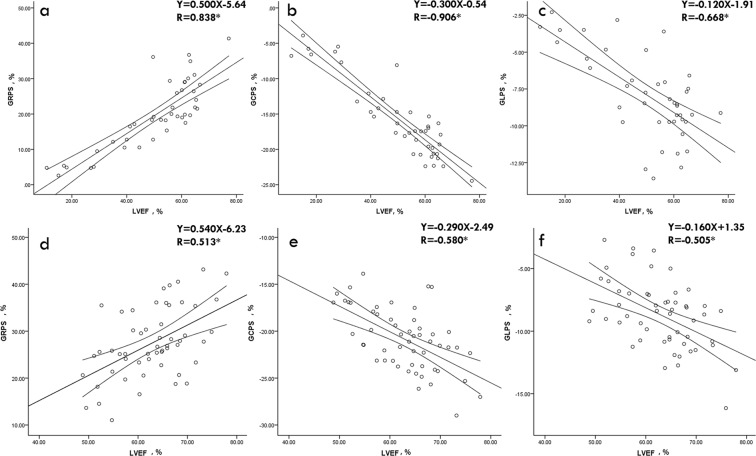
Pearson’s correlation analysis of LVEF with GRPS, GCPS, and GLPS in the non-IIM group (**a**–**c**) and IIM group (**d**–**f**). Notes: *P < 0.05. Actual *p* value and 95% CI are provided from (**a**–**f**) as follows: a (95% CI: 0.717, 0.910, *p* < 0.001), b (95% CI: −0.949, −0.831, *p* < 0.001), c (95% CI: *−*0.808, −0.457, *p* < 0.001), (**d**) (95% CI: 0.284, 0.686*, p* < 0.001), (**e**) (95% CI: −0.734, −0.370, *p* < 0.001*)*, and (**f**) (95% CI: −0.681, −0.274, *p* < 0.001). Abbreviations as in Table 1 and Fig. 1.

LVEF was correlated with GRPS (r = 0.838, *p* < 0.001), GCPS (r = −0.906, *p* < 0.001), and GLPS (r = −0.668, *p* < 0.001) in the non-IIM group. In the IIM group, LVEF was also associated with GRPS (r = 0.513, *p* < 0.001), GCPS (r = −0.580, *p* < 0.001), and GLPS (r = −0.505, *p* < 0.001) (see Fig. 2). There were significant correlations between LVEF and GRPS (r = 0.453, p = 0.012), GCPS (r = −0.464, p = 0.010), and GLPS (r = −0.444, p = 0.014) in the normal control subjects (see Supplementary Fig. S2d−f).

### Reproducibility of tissue tracking to assess LV deformation

As shown in Table 3, there were excellent inter-and intra-observer agreements in LV global myocardial PS [intraclass correlation coefficient (ICC), ICC = 0.94–0.98 and 0.97–0.99, respectively]. For the PS of the LV regional segments, the inter- and intra-observer ICC values were 0.89–0.99 and 0.91–0.99, respectively (see Supplementary Figs. S3 and S4).

**Table 3:** Intra- and inter-observer variability of cardiac MRI tissue tracking.

	Inter−observer (n = 30)		Intra−observer (n = 30)	
	ICC	95% CI	ICC	95% CI
**Global**				
Radial PS(%)	0.98	0.95−0.99	0.98	0.97−0.99
Circumferential PS(%)	0.98	0.96−0.99	0.99	0.98−0.99
Longitudinal PS(%)	0.94	0.87−0.97	0.97	0.93−0.98
**Basal**				
Radial PS(%)	0.96	0.93−0.98	0.97	0.93−0.99
Circumferential PS(%)	0.96	0.88−0.99	0.96	0.92−0.98
Longitudinal PS(%)	0.90	0.79−0.95	0.93	0.86−0.97
**Mid**				
Radial PS(%)	0.96	0.91−0.98	0.96	0.91−0.98
Circumferential PS(%)	0.99	0.98−0.99	0.99	0.98−0.99
Longitudinal PS(%)	0.89	0.79−0.95	0.95	0.90−0.98
**Apical**				
Radial PS(%)	0.93	0.86−0.97	0.94	0.88−0.97
Circumferential PS(%)	0.97	0.94−0.99	0.98	0.96−0.99
Longitudinal PS(%)	0.89	0.78−0.94	0.91	0.82−0.96

Notes: ICC, intraclass correlation coefficient; CI, confidence interval; PS, peak strain; MRI, magnetic resonance imaging.

## Discussion

Cardiac involvement in patients with CTD is mostly subclinical and may lead to cardiac-related death due to myocarditis, myocardial fibrosis, valve disorders, coronary vasculitis, and pericarditis^3,6^. The underlying mechanisms of myocardial involvement in CTD are governed by autoimmunity and chronic inflammation^3^. Although the LVEF is conventionally used as a measurement of global cardiac function, this parameter has limited sensitivity for the detection of subclinical conditions^11^. Several studies have shown subclinical impairment of the myocardium in CTD patients with preserved LVEF by speckle tracking echocardiography (STE)^12–14^. While, there exists accumulating evidence emphasizing the role of cardiac MRI tissue tracking in overcoming the shortcomings of low tissue resolution and dependency of an acoustic window in STE for the evaluation of myocardial strain and cardiac dysfunction^15^.

In the present study, we applied cardiac MRI tissue tracking to evaluate LV myocardial systolic strain in patients with CTD and compared LV deformation between IIM and non-IIM subgroups. The main findings were: (1) patients with CTD had impaired global and regional LV systolic strain in the three directions, even though the LVEF was preserved; (2) compared with normal controls, the damaged strain in IIM patients mainly involved GLPS and longitudinal PS at the apical slice, whereas all strain values were impaired in non-IIM patients; (3) with the exception of GLPS and longitudinal PS at the apical slice, the magnitude of all strain parameters in non-IIM patients were lower than those in IIM patients; and (4) GPRS and GCPS showed correlations with LVEF and NT-proBNP level in both IIM and non-IIM patients.

The LV wall is composed of cardiomyocytes, of which the orientation continuously rotates from epicardium to endocardium^16^. Recently, CMR tissue tracking has been gradually used to quantitatively measure LV function via different strain parameters from the radial, circumferential, and longitudinal directions^8,17,18^ with high sensitivity and reproducibility^19,20^. One of the findings of the present study was that, compared with healthy controls, the magnitude of global and regional PS in the three directions was decreased in CTD patients with preserved LVEF, which is consistent with STE results^12,13^. This finding may assist the explanation of the impairment of LV deformation detected by cardiac MRI tissue tracking prior to conventional LVEF in patients with CTD. Another finding was that GRPS, GCPS, and GLPS were correlated with LVEF in patients with CTD, which is similar to the previous literature reports in other diseases^7,21^.

An increasing number of studies have shown subclinical cardiac dysfunction in IIM patients detected by STE^22,23^. In our research, IIM patients with preserved LVEF were found to have impaired strain manifested as the magnitude reduction in GLPS and longitudinal PS at the apical slice, suggesting subclinical LV myocardial systolic impairment. Previous literature has been reported that IIM patients showed impaired LV myocardial microvascular dysfunction^24,25^, and that the abnormal LV myocardial deformation was associated with microvascular dysfunction in other diseases^8,26^. Thus, we presume that the magnitude reduction in longitudinal myocardial strain of IIM patients in our present study may be related to microvascular ischemia. Analogous to our results, recent studies have reported that IIM patients with preserved LVEF have decreased global longitudinal strain^10,23^. Guerra *et al*.^22^ described impaired longitudinal strain involved in the basal and mid-segments detected by STE, which is not consistent with our findings in the apical segment. The reasons for this discrepancy might be related to the difference in techniques used to measure LV deformation or the heterogeneity of patient populations. In addition, compared with healthy subjects, the magnitude of global and regional PS in the three directions was decreased in non-IIM patients with preserved LVEF, which is in agreement with previous reports demonstrating subclinical cardiac systolic dysfunction in other CTD besides IIM, such as SLE, RA, and SSc, as detected by STE^12,13,27^.

Our research revealed that the magnitude of all strain values in non-IIM patients were lower than those in IIM patients (except for GLPS and longitudinal PS at the apical slice). Taken together, these findings support that impairment of LV deformation is different between IIM and non-IIM patients. We presume that myocardial damage manifesting as myocardial perfusion dysfunction and fibrosis differ between IIM and non-IIM. Thus, further studies are required to explore the relationships between LV deformation and the aforementioned myocardial damage detected using multi-parametric cardiac MRI, such as first-pass perfusion and late gadolinium enhancement (LGE) in IIM and non-IIM. In our present study, there was no difference between IIM patients and non-IIM patients in GLPS, and this finding might be contributed to explain GLPS was relative poor parameter (AUC:0.55; specificity: 33.93%) to differentiate IIM patients from non-IIM patients. Furthermore, the data obtained from the ROC analysis supported that the combination of GRPS and GCPS might be a better parameter than any one alone to differentiate IIM patients from non-IIM patients with a relative high sensitivity and specificity.

NT-proBNP is a biologically inactive N-terminal fragment of the active hormone BNP, which is secreted by the myocardium when stimulated by an increase in ventricular overload, ventricular wall stretchesor stress, and is a standard marker of myocardial damage^28^. Myocardial cell injury induced by persistent systemic inflammation and immune dysfunction could increase NT-proBNP level in CTD^28,29^. In the present study, NT-proBNP in non-IIM patients was higher than that in IIM patients, suggesting that the severity of myocardial damage might be different between IIM and non-IIM. Another finding demonstrated that NT-proBNP level was correlated with the global strain parameters in the three directions, which might indicate that LV global deformation is related to myocardial injury in patients with CTD. Similar to our findings, NT-proBNP has been reported to be increased in patients with CTD and correlated with subclinical cardiac disease^30^.

The limitations of this study include the following: first, this was a retrospective and single center study, and potential center-specific bias cannot be excluded. Second, LGE data was not involved in our present study and the relationship between LV deformation and myocardial damage detected by cardiac MRI first-pass perfusion or LGE technologies is not known; thus, further studies are required to explore this. Finally, although cardiac MRI tissue tracking demonstrated high reproducibility, the accuracy needs to be further verified because of the absence of a reference standard.

In conclusion, patients with CTD showed impaired LV deformation detected by cardiac MRI tissue tracking, even though they had preserved LVEF. The impairment of LV deformation was different between IIM and non-IIM patients. Early detection of subclinical impaired LV deformation may help to screen high-risk patients with CTD for early treatment.

## Materials and Methods

### Study population

The study cohort retrospectively enrolled 121 patients with CTD at our hospital from January 2015 to January 2019. CTD was diagnosed according to the criteria of the American College of Rheumatology or the European League Against Rheumatism, respectively. The exclusion criteria included coronary artery disease, cardiomyopathy, congenital heart disease, heart valve disease, and contraindication for cardiac MRI. Finally, 98 patients with CTD (mean age, 45.2 ± 13.2 years; 24 men) were eligible for the study, including 56 patients with IIM and 42 patients with non-IIM [13 with overlap syndrome, 5 with mixed connective tissue disease,12 with SLE, 4 with RA, 4 with Sjogren’ssyndrome,2 with undifferentiated connective tissue disease, and 2 with SSc]. Considering the variety of CTD, these patients were divided into two subgroups: the IIM group (n = 56) and the non-IIM group (n = 42). A total of 30 age- and gender-matched healthy volunteers (mean age, 45.5 ± 12.3 years; 11 men) with no history of cardiovascular or systematic disease were included as the normal controls. All participants underwent cardiac MRI scanning and were examined for the clinical marker, NT-proBNP. The study protocol was approved by the West China Hospital of Sichuan University Biomedical Research Ethics Committee and conducted in an accordance with the ethical guidelines of the Declaration of Helsinki (2013 EDITION)^31^. Informed consent was obtained from all subjects.

### Cardiac MRI protocol

All patients were examined using a 3.0T whole-body scanner with a 32-channel phase-array cardiovascular coil (Trio Tim; Siemens Medical Solutions, Erlangen, Germany). All participants were examined in the supine position. The breath-hold technique and a manufacturer’s standard electrocardiographic gating device were used for monitoring the participant’s breathing and electrocardiogram values, respectively. The continuous data were acquired during the breath-holding period. A series of 8–12 continuous cardiac MRI cine sections were acquired in the short-axis from the mitral valve level to the LV apex using a balanced steady state free precession (bSSFP) sequence (TR/TE: 39.34/1.22 ms, flip angle: 40°, slice thickness: 8 mm, field of view: 250 × 300 mm, and matrix size: 208 × 139, frequency encode direction: R-L, phase encode direction: A-P and perpendicular to the direction of blood flow). The cardiac cine series in the long-axis two-, three-, and four-chamber views were also obtained.

### Cardiac MRI image analysis

**Figure 3:**
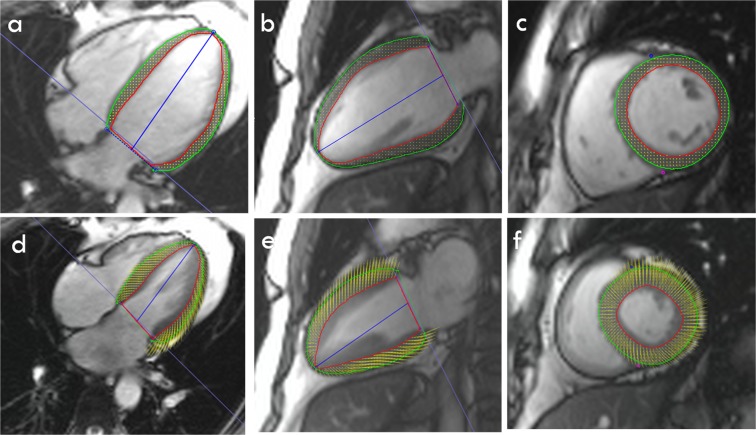
Cardiac MRI tissue tracking in the four-chamber long-axis, two-chamber long-axis and short-axis cine images at the end-diastole (**a**–**c**) and end-systole (**d**–**f**). The red and green curves show the endocardial and epicardial borders, respectively; the yellow dots represent the myocardial voxel points. Abbreviations: MRI, magnetic resonance imaging.

Post-processing of all images was performed offline by two experienced radiologists using commercial software (cvi42, version 5.9.3; Circle Cardiovascular Imaging Inc., Calgary, AB, Canada). The LV functional parameters, including LVEDV, LVESV, LVSV, and LVEF were calculated using the aforementioned software following manual contouring of the endocardial and epicardial borders at the end-diastolic and end-systolic periods of the short-axis cine images with the papillary muscles and moderator bands included. Reduced LVEF was defined as <50%^32^. LV myocardial strain measurement was performed by loading short-axis, long-axis two- and four-chamber slices into the three-dimensional tissue tracking module^33^. All series of the endocardial and epicardial borders were outlined manually in each slice at the end-diastolic period (reference phase) (see Fig. 3). With caution, the papillary muscles and moderator bands were excluded. Conforming to AHA standard regional division for LV^34^, strain in basal, mid-ventricular and apical slice was analysed. The global and regional (basal, middle, and apical slice) strain parameters, including the myocardial PS in the radial, circumferential, and longitudinal directions, were obtained automatically using the aforementioned software.

### Reproducibility of tissue tracking

Intra-observer variability was assessed by comparing the tissue tracking measurements obtained randomly among 30 individuals by the same observer at an interval of 2 weeks. Inter-observer variability was also obtained randomly among 30 individuals and was determined by comparing the independent tissue tracking measurements of two experienced and double-blinded observers.

### Statistical analysis

Statistical analysis was performed using IBM SPSS Statistics for Windows (version 24.0; IBM Corporation, Armonk, NY, USA). All continuous variables were tested for normality using the Kolmogorov–Smirnov test. The homogeneity of variance was assessed using Levene’s test. Continuous variables are expressed as the mean ± standard deviation (SD) or the median (interquartile range). LV function and strain parameters between normal controls and CTD groups were compared using a Student’s *t-*test or Mann–Whitney U test, whereas those parameters among normal controls, IIM and non-IIM subgroups were compared by one-way repeated analysis of variance (ANOVA) or the Kruskal–Wallis rank test, as appropriate. Least-Significant-Difference test was used for multiple pairwise comparisons among the three groups of normal controls, IIM group, and non-IIM group when the *p*-value of one-way ANOVA was less than 0.05. Pearson’s correlation was used to evaluate the relationship between strain parameters and NT-proBNP and LVEF. ROC analysis was performed to determine whether the global strain parameters can be used to differentiate IIM from non-IIM patients. Intra- and inter-observer variabilities for reproducibility were assessed using the ICC. A two-tailed *p* value of < 0.05 was considered statistically significant.

## Supplementary information

Left Ventricular Deformation in Patients with Connective Tissue Disease: Evaluated by 3.0T Cardiac Magnetic Resonance Tissue Tracking

## Data Availability

The datasets used during the current study are available from the corresponding author on reasonable request.
