# Supplementary material for: Left Ventricular Deformation in Patients with Connective Tissue Disease: Evaluated by 3.0T Cardiac Magnetic Resonance Tissue Tracking
Source: Sci Rep. 2019 Nov 29;9:17913. doi: 10.1038/s41598-019-54094-1 (PMC6884516; doi:10.1038/s41598-019-54094-1)
Supplement: Supplementary file 1 — Left Ventricular Deformation in Patients with Connective Tissue Disease: Evaluated by 3.0T Cardiac Magnetic Resonance Tissue Tracking [file 41598_2019_54094_MOESM1_ESM.zip › Supplementary Information/supplementary information.pdf]

Supplementary Information:

Left Ventricular Deformation in Patients with Connective Tissue Disease: Evaluated by 3.0T Cardiac Magnetic Resonance Tissue Tracking

Jin Wang[1]†, MD; Ke Shi[1]†, MD; Qin Zhao[1], MD; Zhi-gang Yang[1]*, MD, PhD; Ying-kun Guo[2]*, MD; Hua-yan Xu[2] MD; Xi Liu[1], MD; Yue Gao[1], MD; Hong Yu[1], MD

[1]Department of Radiology, West China Hospital, Sichuan University, 37# Guo Xue Xiang, Chengdu, Sichuan 610041, China.

[2]Department of Radiology, Key Laboratory of Birth Defects and Related Disease of Woman and Children of Ministry of Education, West China Second University Hospital, Sichuan University, Chengdu, China.

†Jin Wang and Ke Shi contributed equally to this work.

*Zhi-gang Yang and Ying-kun Guo contributed equally to this work.

Figure Legends :

Supplementary Fig. S1. Comparison of LV strain parameters among normal controls, IIM patients and non-IIM patients. Notes: $^{*}P < 0.017$ versus normal group; $^{\S}P < 0.017$ versus patients with IIM. IIM, idiopathic inflammatory myopathy; PS: peak strain; GRPS, global radial PS; GCPS, global circumferential PS; GLPS, global longitudinal PS; BRPS, radial PS at the basal slice ; BCPS, circumferential PS at the basal slice; BLPS, longitudinal PS at the basal slice; MRPS, radial PS at the mid slice ; MCPS, circumferential PS at the mid slice; MLPS, longitudinal PS at the mid slice; ARPS, radial PS at the apical slice ; ACPS, circumferential PS at the apical slice; ALPS, longitudinal PS at the apical slice.

Supplementary Fig. S2. Pearson's correlation analysis of Nt-proBNP level with GRPS, GCPS, and GLPS (a–c) and the relationship between LVEF and GRPS, GCPS, and GCPS (d–f) in the normal control subjects. Notes: $^{*}P < 0.05$. Actual *p* value and 95% CI are provided from a-f as follows: a (95% CI: –0.223, 0.483, $p = 0.432$), b (95% CI: –0.322, 0.397, $p = 0.820$), and c (95% CI: –0.542, 0.147, $p = 0.231$), d (95% CI: 0.110, 0.700, $p = 0.012$), e (95% CI: –0.706, –0.125, $p = 0.010$), and f (95% CI: –0.694, –0.100, $p = 0.014$). LVEF, LV ejection fraction; NT-proBNP, N-terminal pro-brain natriuretic peptide; GRPS, global radial peak strain; GCPS, global circumferential peak strain; GLPS, global longitudinal peak strain; IIM, idiopathic inflammatory myopathy.

Supplementary Fig. S3. Bland–Altman plots with limits of agreement (95% confidence intervals) demonstrating the intra-observer and inter-observer reproducibility of CMR myocardial tissue tracking strain parameters of global and at the basal slice. Notes: Solid lines represent bias (blue) and 95% limits of agreement (red). Abbreviations as in Fig. S1.

Supplementary Fig. S4. Bland–Altman plots with limits of agreement (95% confidence intervals) demonstrating the intra-observer and inter-observer reproducibility of CMR myocardial tissue tracking strain parameters of the mid and apical slices. Notes: Solid lines represent bias (blue) and 95% limits of agreement (red). Abbreviations as in Fig. S1.
