# Supplementary figures and images for: Left Ventricular Deformation in Patients with Connective Tissue Disease: Evaluated by 3.0T Cardiac Magnetic Resonance Tissue Tracking
Source: Sci Rep. 2019 Nov 29;9:17913. doi: 10.1038/s41598-019-54094-1 (PMC6884516; doi:10.1038/s41598-019-54094-1)

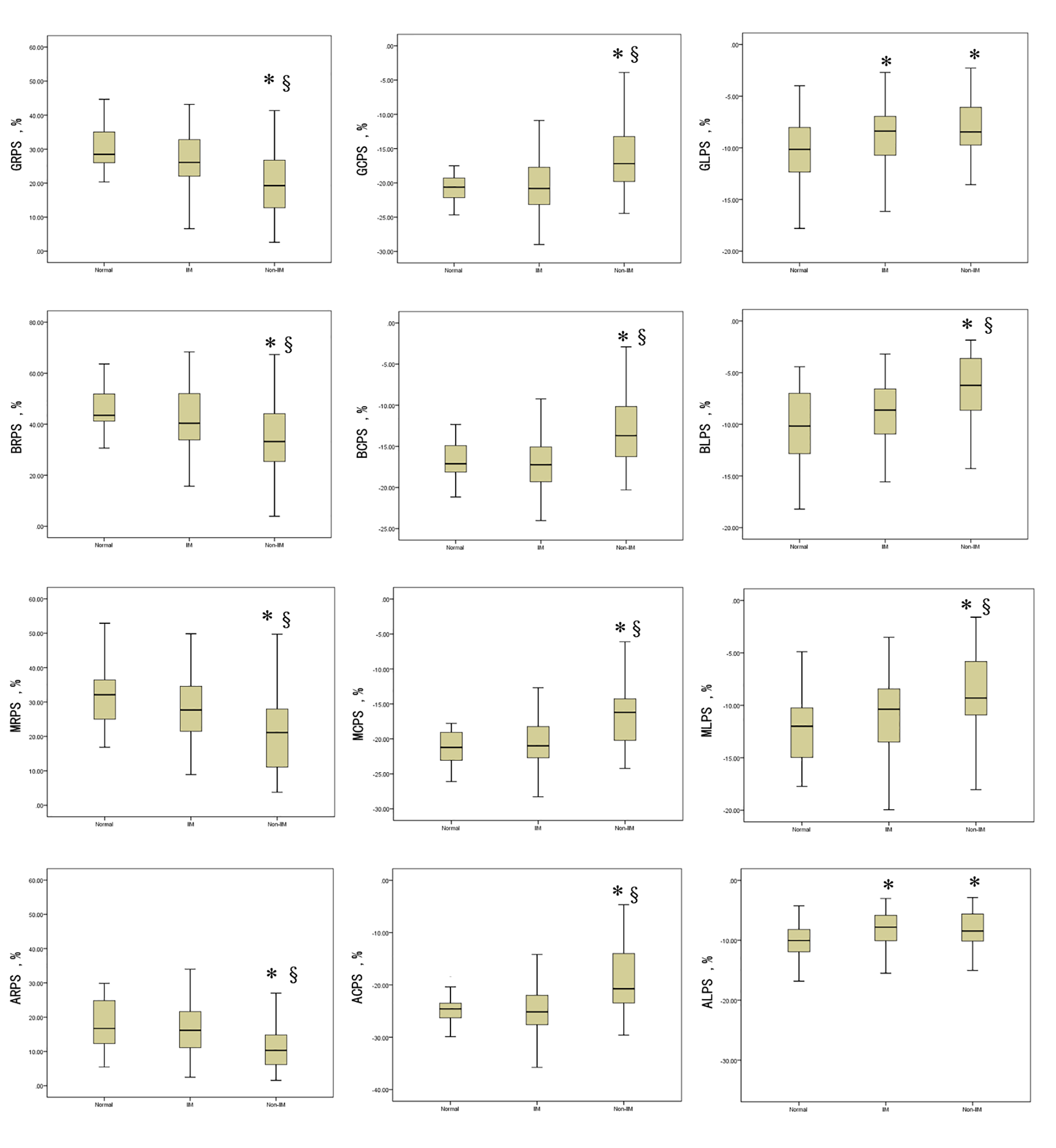

Supplement: Supplementary file 1 — Left Ventricular Deformation in Patients with Connective Tissue Disease: Evaluated by 3.0T Cardiac Magnetic Resonance Tissue Tracking [file 41598_2019_54094_MOESM1_ESM.zip › Supplementary Information/Supplementary Fig. S1.tif]

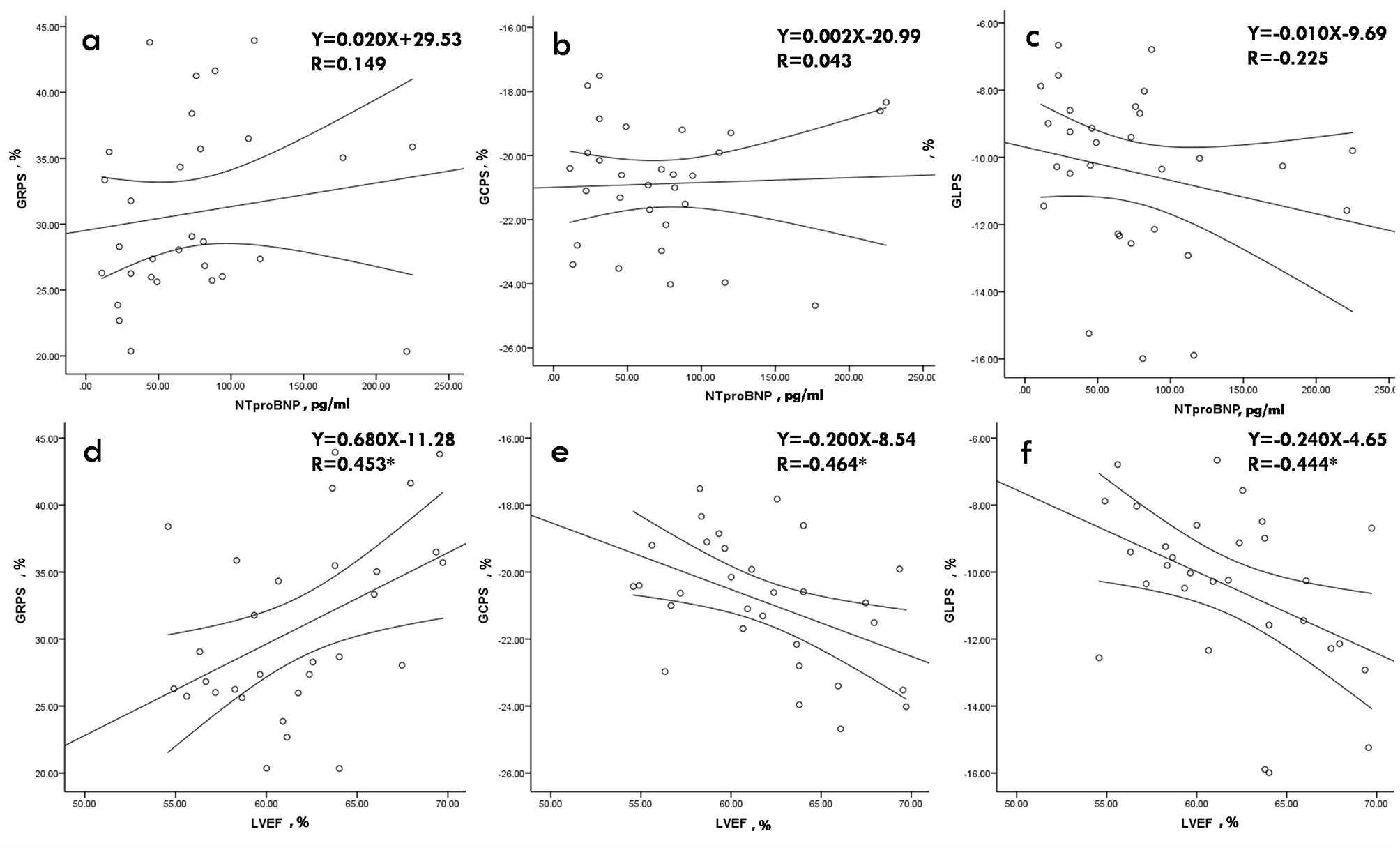

Supplement: Supplementary file 1 — Left Ventricular Deformation in Patients with Connective Tissue Disease: Evaluated by 3.0T Cardiac Magnetic Resonance Tissue Tracking [file 41598_2019_54094_MOESM1_ESM.zip › Supplementary Information/Supplementary Fig. S2.tif]

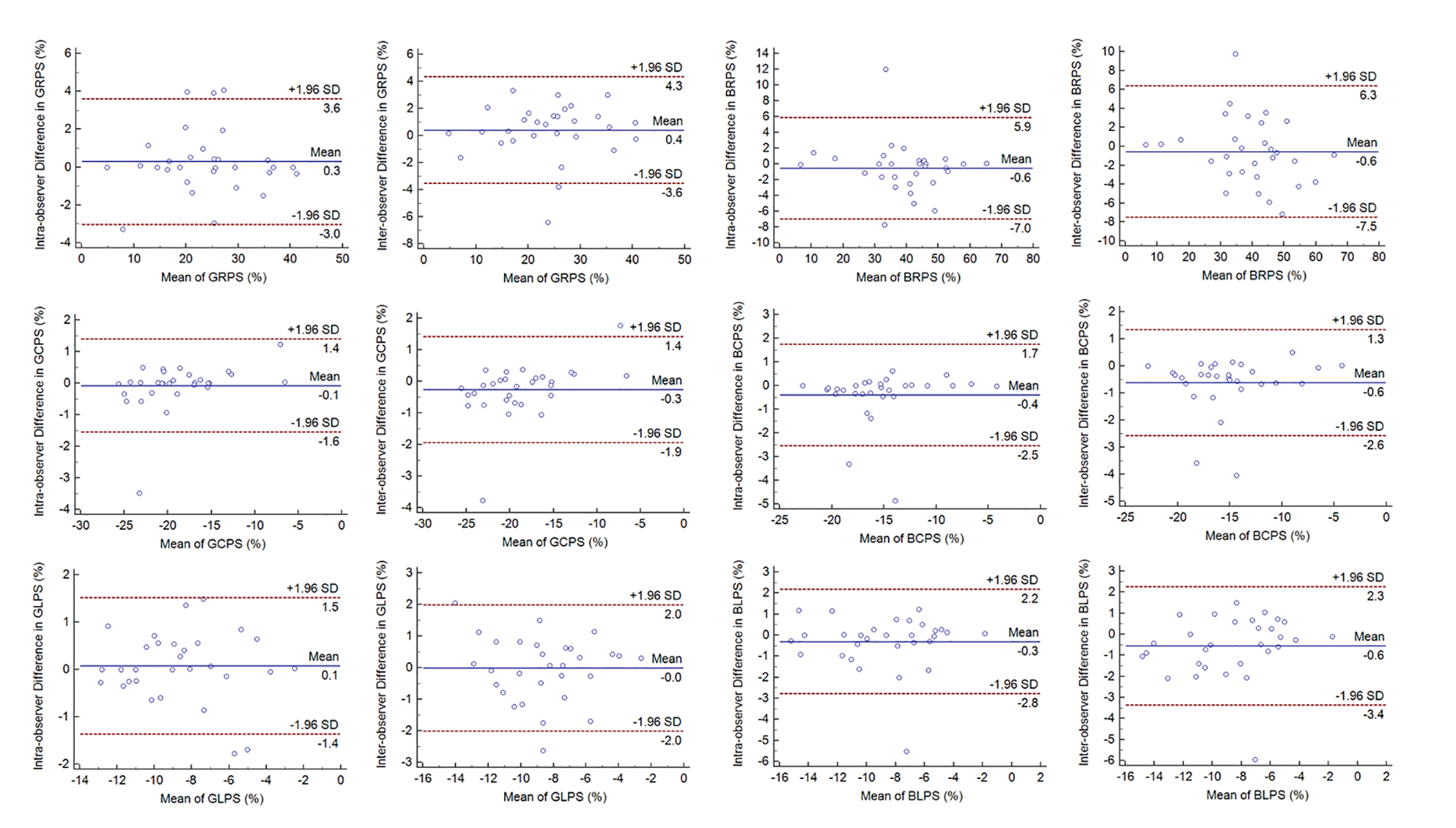

Supplement: Supplementary file 1 — Left Ventricular Deformation in Patients with Connective Tissue Disease: Evaluated by 3.0T Cardiac Magnetic Resonance Tissue Tracking [file 41598_2019_54094_MOESM1_ESM.zip › Supplementary Information/Supplementary Fig. S3.tif]

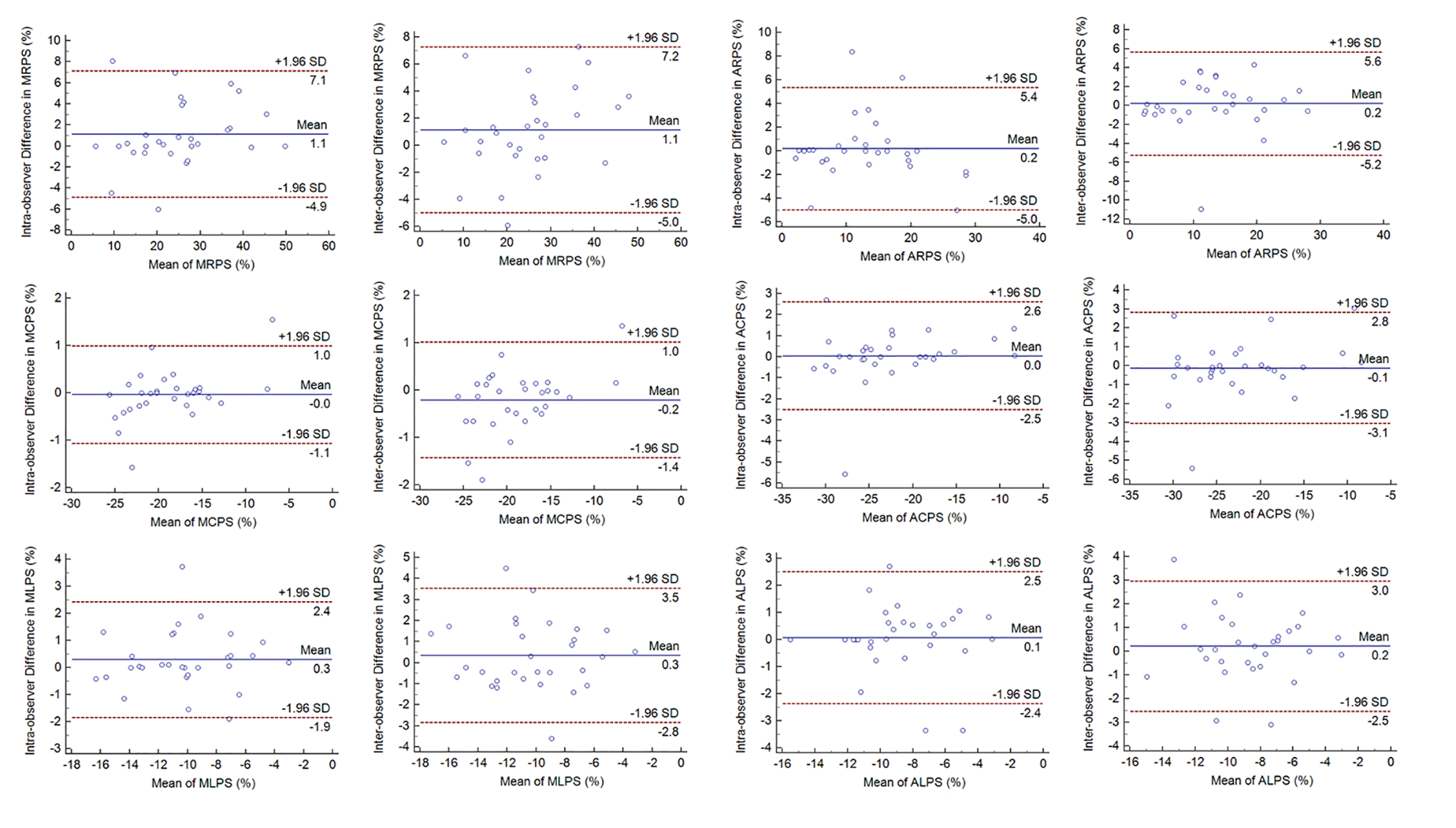

Supplement: Supplementary file 1 — Left Ventricular Deformation in Patients with Connective Tissue Disease: Evaluated by 3.0T Cardiac Magnetic Resonance Tissue Tracking [file 41598_2019_54094_MOESM1_ESM.zip › Supplementary Information/Supplementary Fig. S4.tif]
